# Free and Nanoencapsulated Tobramycin: Effects on Planktonic and Biofilm Forms of *Pseudomonas*

**DOI:** 10.3390/microorganisms5030035

**Published:** 2017-06-26

**Authors:** Eulalia Sans-Serramitjana, Marta Jorba, Ester Fusté, José Luis Pedraz, Teresa Vinuesa, Miguel Viñas

**Affiliations:** 1 Laboratory of Molecular Microbiology and Antimicrobials, Department of Pathology and Experimental Therapeutics, Faculty of Medicine & Health Sciences, University of Barcelona, 08007 Barcelona, Spain; eulalia.bio.87@gmail.com (E.S.-S.); m.jorba.pedrosa@gmail.com (M.J.); esterfustedominguez@ub.edu (E.F.); tvinuesa@ub.edu (T.V.); 2 School of Nursing, Faculty of Medicine & Health Sciences, University of Barcelona, 08007 Barcelona, Spain; 3 Laboratory of Pharmaceuticals, University of the Basque Country and Biomedical Research Networking Center in Bioengineering, Biomaterials and Nanomedicine (CIBER-BBN), 48940 Lejona, Spain; joseluis.pedraz@ehu.es

**Keywords:** tobramycin, lipid nanoparticles, antibacterial and antibiofilm effects, *P. aeruginosa*, cystic fibrosis

## Abstract

Cystic fibrosis (CF) is a genetic disorder in which frequent pulmonary infections develop secondarily. One of the major pulmonary pathogens colonizing the respiratory tract of CF patients and causing chronic airway infections is *Pseudomonas* *aeruginosa*. Although tobramycin was initially effective against *P. aeruginosa*, tobramycin-resistant strains have emerged. Among the strategies for overcoming resistance to tobramycin and other antibiotics is encapsulation of the drugs in nanoparticles. In this study, we explored the antimicrobial activity of nanoencapsulated tobramycin, both in solid lipid nanoparticles (SLN) and in nanostructured lipid carriers (NLC), against clinical isolates of *P. aeruginosa* obtained from CF patients. We also investigated the efficacy of these formulations in biofilm eradication. In both experiments, the activities of SLN and NLC were compared with that of free tobramycin. The susceptibility of planktonic bacteria was determined using the broth microdilution method and by plotting bacterial growth. The minimal biofilm eradication concentration (MBEC) was determined to assess the efficacy of the different tobramycin formulations against biofilms. The activity of tobramycin-loaded SLN was less than that of either tobramycin-loaded NLC or free tobramycin. The minimum inhibitory concentration (MIC) and MBEC of nanoencapsulated tobramycin were slightly lower (1–2 logs) than the corresponding values of the free drug when determined in tobramycin-susceptible isolates. However, in tobramycin-resistant strains, the MIC and MBEC did not differ between either encapsulated form and free tobramycin. Our results demonstrate the efficacy of nanoencapsulated formulations in killing susceptible *P. aeruginosa* from CF and from other patients.

## 1. Introduction

Cystic fibrosis (CF) is the most common genetic disorder in the Caucasian population and it is characterized by a high morbidity and mortality. The CF lung is compromised by the production of viscous mucus secretions, resulting in a debilitated mucociliary clearance that promotes bacterial infection and inflammation [1]. *Pseudomonas aeruginosa* is the predominant opportunistic pathogen infecting the respiratory tract of CF patients. Once chronic lung colonization occurs, *P. aeruginosa* changes phenotypically to produce alginate, which allows the bacterium to become established within mucoid biofilms and thus highly resistant to multiple antimicrobials [2,3]. Indeed, the emergence of multidrug-resistant phenotypes and treatment failure were shown to correlate with the reduced permeability of the outer membrane of *P. aeruginosa* to most antimicrobials and the acquisition of genes, encoding antimicrobial resistance [4,5].

Tobramycin is a hydrophilic, cationic antibiotic administered as an aerosol in the treatment of *P. aeruginosa* lung infections in CF patients [6]. Like other aminoglycosides, tobramycin targets the bacterial ribosome, such that bacterial resistance, although rare, mainly involves impermeability and the acquisition of aminoglycoside-modifying enzymes, encoded either on a plasmid or within the genome by transposable elements [7]. However—despite their chemical stability, fast bactericidal effect, synergy with ß-lactam antibiotics, and low incidence of resistance—aminoglycosides are of limited utility because of their nephrotoxicity [8,9]. While tobramycin is less nephrotoxic than gentamicin and other aminoglycosides and has been successfully used against *P. aeruginosa* [10], planktonic bacteria are much more sensitive than bacteria in biofilms, the growth form occurring in the lower respiratory tract of CF patients. The reduced efficacy of tobramycin and other antimicrobials is due to poor mucus penetration, the resilience of the extracellular matrix of the biofilm, and inactivation of the drug through various binding interactions in the infected CF lung [11]. The mechanisms of tobramycin resistance are, to our knowledge, not fully understood. The relationship between mucoidity and tobramycin resistance has been explored; the main conclusion is that mucoidity per se has no effect on resistance [12]. However, the study also distinguished between the roles of mucoidity and biofilm formation in the ability of *P. aeruginosa* to resist antibiotic treatment. While biofilm formation expectantly increased resistance of PAO1 to tobramycin, uncontrolled alginate production did not. Thus, one should speculate that other mechanisms than external matrix have to be involved in the resistance caused by biofilm. It has been shown that iron regulation clearly affects susceptibility, but also gene expression differences.

Nanoformulations such as lipid nanoparticles could improve the delivery of tobramycin and thus enhance its activity. Lipid nanoparticles with a solid matrix are available as solid lipid nanoparticles (SLN) and as newer-generation lipid nanostructured lipid carriers (NLC). SLN are composed of solid lipids. NLC are prepared from a blend of a solid lipid with a liquid lipid. Both are stabilized by surfactants and are able to incorporate lipophilic and hydrophilic drugs [13].

Among the key benefits of lipid nanoparticles in the pulmonary delivery of antibiotics are the improved bioavailability and rapid distribution of the drug; precise targeting of the site of infection; the need for a lower dose; longer administration intervals, thereby reducing the risk of serious dose-related side effects; and the scaling-up feasibility of nanoparticle production [14]. The specific advantages of SLN and NLC over other delivery systems include their higher stability compared to liposomes, both in vitro and in vivo [13,15], as well as their better biocompatibilities and lower potential toxicity (both acute and chronic) compared to polymeric nanoparticles and other synthetic formulations [15,16]. Moreover, the use of nanoparticles could overcome pre-existing drug resistance mechanisms, including those involving the decreased uptake and increased efflux of the drug, to achieve better biofilm penetration. Previous studies testing the effectiveness of aminoglycosides against clinical isolates of *P. aeruginosa* reported better results with compounds loaded in nanoformulations than with the free drug [17,18,19].

Based on these findings and our own results demonstrating the antimicrobial activity of colistin loaded into lipid nanoparticles [20,21], in this work we explored the activity of nanoencapsulated (both SLN and NLC) tobramycin versus that of the free drug against *P. aeruginosa* clinical isolates obtained from CF patients. We then investigated the efficacy of these novel formulations in the eradication of *P. aeruginosa* biofilms. The main purpose was to demonstrate that after their inclusion in nanoparticles tobramycin was able to conserve its antimicrobial activity. Even when antimicrobial action is not higher, the nanoparticles are of interest since pharmacology has demonstrated a better distribution in the respiratory tree of molecules in lipid nanoparticles. Moreover, we have reported that in vivo nanoparticles spread homogenously through the lung and there is no migration of lipid nanoparticles to other organs, such as liver, spleen, or kidneys [21].

## 2. Materials and Methods

### 2.1. Bacterial Isolates

The 34 clinical isolates of *P. aeruginosa* (17 non-mucoid and 17 mucoid) included in this study were obtained from the sputum samples and pharyngeal exudates of CF patients seen at the University Hospital Vall d’Hebrón and University Hospital Sant Joan de Déu (Barcelona, Spain) between January and April 2012. The patients (59% female, 41% male) ranged in age from 9 to 50 years (mean: 27 years). *P. aeruginosa* strains ATCC 27853 and PAO1 served as the control strains in the drug susceptibility assays and biofilm studies, respectively. Two CF clinical isolates, *P. aeruginosa* strain 362VH (tobramycin-resistant) and strain 056SJD (tobramycin-susceptible), were used to evaluate the ability of free and nanoencapsulated tobramycin to inhibit bacterial growth and eradicate bacterial biofilms. Table 1 summarizes the isolates used and their main characteristics.

### 2.2. Chemicals and Bacteriological Media

Tobramycin was purchased from Sigma-Aldrich Chemicals (St. Louis, MO, USA). Mueller-Hinton II broth cation-adjusted (MHBCA) was from Becton Dickinson (Sparks, MD, USA). Tryptone soy agar (TSA) was purchased from Sharlau (Sentmenat, Barcelona, Spain). Precirol ATO 5 was kindly provided by Gattefossé (Madrid, Spain), and poloxamer 188 by BASF (Ludwigshafen, Rhineland-Palatinate, Germany). Polysorbate and Tween 80 were purchased from Panreac Química (Castellar del Vallès, Barcelona, Spain). Miglyol 812 was provided by Sasol (Hamburg, Germany).

### 2.3. Preparation of Lipid Nanoparticles

Tobramycin-loaded nanoparticles were prepared as described. Briefly, two loaded formulations were elaborated, namely solid lipid nanoparticles (SLN) and nanostructured lipid carriers (NLC) [21]. An emulsion solvent evaporation technique was chosen for the preparation of SLN. Briefly, 10 mg of antibiotic (Sigma-Aldrich, St. Louis, MO, USA) were mixed with a 5% (*w*/*v*) Precirol^®^ ATO 5 (Gattefossé, Madrid, Spain) dichloromethane solution. Then, the organic phase and an aqueous surfactant containing solution (Poloxamer 188 at 1% *w*/*v* and Polysorbate 80 at 1% *w*/*v*) were mixed and emulsified by sonication at 20 W for 30 s (Branson Sonifier 250, Danbury, CT, USA). The solvent was allowed to evaporate by magnetic stirring for 2 h at room temperature. Subsequently, the resulting SLNs were washed by centrifugation in Amicon^®^ centrifugal filtration units (100,000 MWCO, Merck Millipore, Billerica, MA, USA) at 2500 rpm for 15 min three times. For the NLC elaboration, a hot melt homogenization technique was selected. In brief, Precirol^®^ ATO 5 and Miglyol^®^ 812 (Sasol, Johannesburg, South Africa) were selected as the lipid core. Those lipids were mixed with the API and heated above the melting temperature of the solid lipid. The surfactant solution consisted of 1.3% (*w*/*v*) of Polysorbate 80 and 0.6% (*w*/*v*) of Poloxamer 188. The lipid and aqueous solutions were heated to the same temperature and then emulsified by sonication for 15 s at 20 W. Nanoparticles were stored at 4 °C overnight to allow lipid re-crystallization and particle formation. Then, a washing step was undergone by centrifugation at 2500 rpm in Amicon^®^ centrifugal filtration units (100,000 MWCO) three times. All the nanoparticles prepared were freeze-dried with two different cryoprotectants, either d-mannitol or trehalose (15%). In SLN formulations, emulsifiers constituted the aqueous phase of the emulsions, stabilizing the lipid dispersion of the nanoparticles and preventing their agglomeration [22]. Thus, the influence of the emulsifier on the bioactivity of the lipid nanoparticles was examined in two different types of SLN. SLN-tobramycin nanoparticles were prepared using the emulsifiers poloxamer 188 and polysorbate 80, each at 1% *w*/*v*. SLN-SDS-tobramycin nanoparticles were prepared using 2% sodium dodecyl sulfate (SDS) as the co-emulsifier. NLCs loaded with tobramycin (NLC-tobramycin) were prepared using a hot melt homogenization technique, following the method described by Pastor et al. [21].

All three types of nanoparticles used in this work (SLN-tobramycin, SLN-SDS-tobramycin, and NLC-tobramycin) were stabilized by trehalose, since in previous research we determined that it was a better cryoprotectant than mannitol [20]. Solid Lipid Nanoparticles and Nanostructured lipid carriers were characterized for size, polidispersity index (PDI) and Z-potential by means of Zetaseiser Nano ZS (Malvern Instruments, Worcestershire, UK). Measurements were based on Dynamic Light Scattering (DLS). Atomic force microscopy images were obtained by using a XE-70 atomic force microscope (Park Systems, Suwon, Korea).

### 2.4. Drug Susceptibility Assay in Planktonic Bacteria

Susceptibility to free tobramycin and to the three formulations of nanoencapsulated tobramycin was determined using the broth microdilution method in accordance with the Clinical Laboratory Standards Institute [23]. Briefly, the isolates were grown overnight at 37 °C in MHBCA, after which 2 mL of the culture was used to inoculate 20 mL of fresh MHBCA medium. After 2 h at 37 °C and 200 rpm, the bacterial cultures were adjusted to an optical density at 625 nm (OD_625nm_) of 0.08–0.1 and diluted 1:1000 in fresh MHBCA medium. Five µL of each diluted suspension was added to the wells (10^4^ UFC/well) of 96-well microtiter plates previously filled with MHBCA and serially diluted antibiotic (free and nanoencapsulated). The plates were incubated at 37 °C for 24 h, after which the minimal inhibitory concentration (MIC) was determined macroscopically, based on the visually assessed turbidity of the wells. All experiments were performed in triplicate with three technical replicates.

### 2.5. Effect of Free and Nanoencapsulated Tobramycin on P. aeruginosa Growth

Two *P. aeruginosa* CF isolates, tobramycin-susceptible strain 056SJD and tobramycin-resistant strain 362VH, were used to examine the effect of free and nanoencapsulated (SLN and NLC) tobramycin. The antimicrobials were added to exponentially growing liquid cultures (1 × 10^8^ CFU/mL, in MHBCA) at concentrations above and below the MIC. Samples were taken aseptically at 0, 1, 2, 3, 4, and 5 h from bacterial cultures incubated at 37 °C with shaking (250 rpm). Bacterial growth was measured optically to determine the OD_625nm_. All measurements were carried out in triplicate.

### 2.6. Antimicrobial Susceptibility of Sessile Bacteria

The minimal biofilm eradication concentration (MBEC), defined in this study as the minimal antibiotic concentration required to eliminated >90% of the non-treated biofilm, was determined as described by Moskowitz et al. [24], with modifications. Briefly, the formation of bacterial biofilms was promoted as follows: the pegs of a modified polystyrene microtiter lid (catalog No. 445497; Nunc TSP system) were immersed into 96-well microtiter plates containing inoculated (10^4^ UFC/well) 200 μL MHBCA/well. The modified plates were left undisturbed at 37 °C for 24 h. The pegs were then gently rinsed in 0.9% NaCl and the bacterial biofilms exposed to different concentrations of free and nanoencapsulated tobramycin for 24 h at 37 °C in MHBCA. The pegs were then rinsed again with 0.9% NaCl and the biofilms removed by 10 min sonication and centrifugation (2000 rpm, 10 min) in a BioSan Laboratory Centrifuge LMC-3000. Bacteria recovered from the biofilms were incubated for 24 h at 37 °C. Pegs were again rinsed with 0.9% NaCl solution and biofilms removed by 10 min sonication. Recovered bacteria were incubated for 24 h at 37 °C. Optical densities at 620 nm were measured in order to determine MBEC values. All experiments were performed in triplicate on at least three occasions.

### 2.7. Statistical Analysis

The antimicrobial susceptibilities of the tested *P. aeruginosa* strains to free and nanoencapsulated tobramycin were statistically analyzed using Cochran’s Q test. A *p*-value < 0.05 was considered to indicate statistical significance.

## 3. Results and Discussion

### 3.1. Nanoparticle Characterization

**Figure 1:**
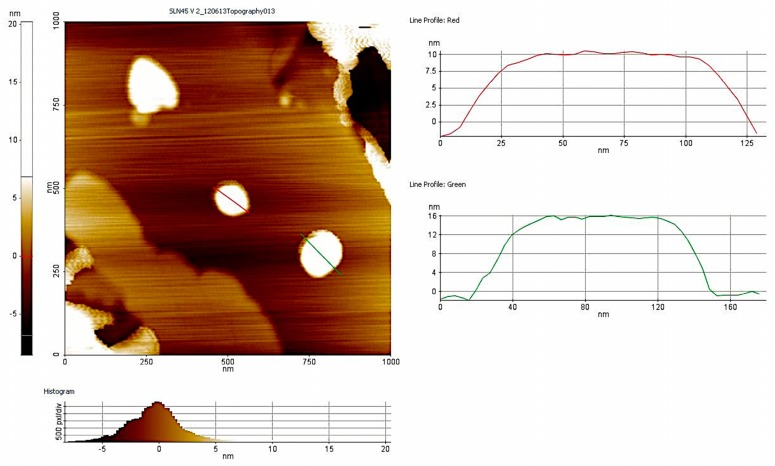
Size measurement performed by AFM imaging. Diameter was around 150 nm.

Main characterization data of nanoparticles are shown in Table 2. AFM imaging and size measurements of particles are presented in Figure 1.

### 3.2. Antimicrobial Activity of Free and Nanoencapsulated Tobramycin

Nearly all the isolates tested in this study resulted to be susceptible (MIC ≤ 4 μg/mL) to both the free and nanoencapsulated tobramycin formulations (Figure 1). The MIC of free tobramycin tested against the isolates was 0.5 μg /mL, whereas that of NLC-tobramycin was slightly lower (between 0.25 μg /mL and 0.5 μg /mL) and was also lower than the MICs of SLN-and SLN-SDS-tobramycin (between 1 and 4 μg /mL and 0.5 μg /mL, respectively) type.

In addition, NLCs were much more active than either of the SLN preparations, as evidenced by MIC values of 0.5 and 1–4 μg /mL (*p* < 0.05), respectively. Among the two types of SLN, the formulation prepared without SDS lost antimicrobial activity (up to eight-fold higher MICs) (Figure 1). Thus, further experiments were conducted using NLC and SLN-SDS.

The efficient antibacterial activity of lipid nanoparticles loaded with tobramycin may be due to their small size and physic-chemical properties, which facilitates diffusion of the drug into the bacterial cell [25]. Similar results were reported by Ghaffari et al. [19] in their study of *P. aeruginosa* clinical isolates obtained from CF patients. The authors showed that tobramycin loaded in lipid nanoparticles had the same or higher antimicrobial activity than the free form of the drug. The slightly higher bioactivity of tobramycin-loaded NLC than SLN can be attributed to the higher drug-loading capacity of these nanoparticles and the avoidance of drug loss during storage [14,26]. As demonstrated by Moreno-Sastre et al. [27], second-generation NLC are more stable than first-generation SLN and they can be stored at a wider range of temperatures without relevant modifications of their antimicrobial activity.

**Figure 2:**
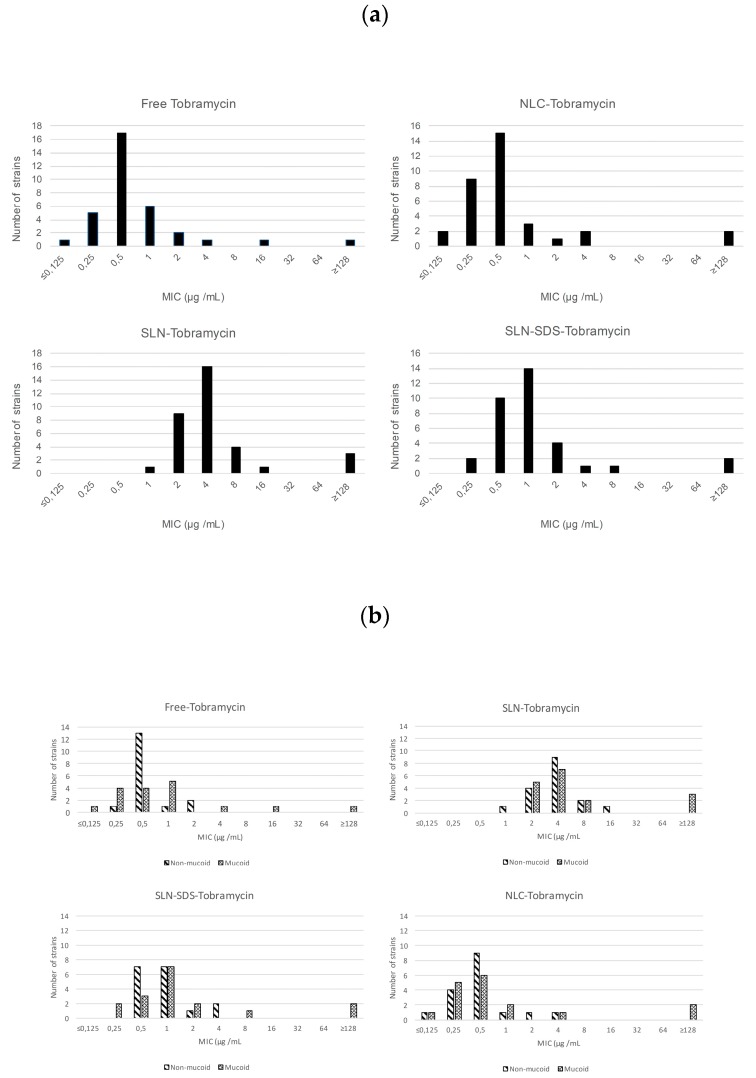
(**a**) The bioactivity (minimum inhibitory concentration, MIC) of lipid nanoparticles loaded with tobramycin in 34 strains of *Pseudomonas aeruginosa* isolated from the clinical samples of cystic fibrosis patients; (**b**) The same as in (**a**) but separated according to the 17 mucoid and 17 non-mucoid strains of the bacterium. For an explanation of the nanoparticles, see the text.

The improved antibacterial activity of SLN-SDS vs. the SLN particles suggests that SDS, when used as a co-emulsifier, confers improved drug stability and release. SDS may also facilitate contact between the lipid nanoparticles and water, resulting in a better distribution equilibrium of the drug. Of relevance to our findings is the major challenge posed by ensuring drug stability in the development of colloidal drug carriers, which offer a high surface area and short diffusion pathways [28]. Figure 2b shows the data separated for mucoid and non-mucoid strains of *P. aeruginosa*.

### 3.3. Effect of Free and Nanoaencapsulated Tobramycin on Bacterial Growth

**Figure 3:**
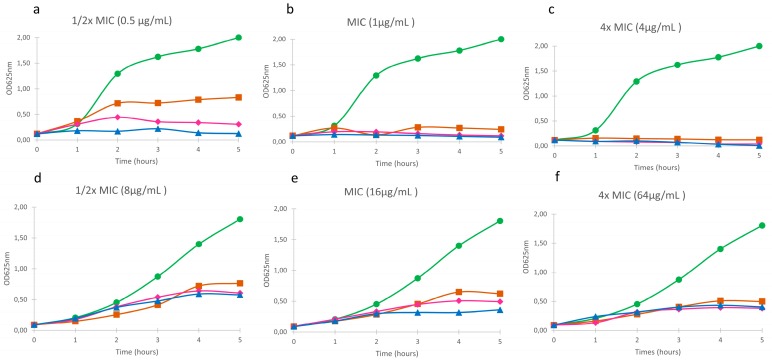
The effect of free and nanoencapsulated (SLN and NLC) tobramycin on the growth of *P. aeruginosa*. (**a**,**b**,**c**) strain 056SJD; (**d**,**e**,**f**) strain 362VH. Control (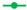); Free tobramycin (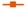); SLN-Tobramycin (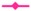); NLC-Tobramycin (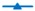).

The susceptibilities of non-mucoid, susceptible (isolate 056SJD) and mucoid, resistant (isolate 362VH) *P. aeruginosa* to free and nanoencapsulated tobramycin were similar at all concentrations of the antibiotic tested (Figure 3). At sub-inhibitory concentrations (1/2 × MIC), the effect of the tobramycin-loaded lipid formulations on the growth kinetics of susceptible isolate was slightly lower than that of the free drug (Figure 3a) whereas the response of the resistant isolate did not differ (Figure 3d). At the MIC, greater inhibition of the susceptible isolate was achieved, since after 5 h of antimicrobial exposure none of the formulations was able to fully inhibit the growth of the resistant isolate (Figure 3b,e). At concentrations above the MIC, the growth of the susceptible isolate was inhibited immediately after the addition of the antimicrobial (Figure 3c), but, again, none of the formulations fully inhibited the growth of the resistant isolate (Figure 3f). Empty lipid nanoparticles had no antibacterial activity in either isolate (data not shown).

Taken together, our results demonstrate that the loading of tobramycin into lipid nanoparticles does not adversely affect the antimicrobial activity of the drug against planktonic *P. aeruginosa*. The preserved potency of lipid nanoparticles containing tobramycin may be due to their facilitated diffusion across the bacterial cell membranes. Mugabe et al. [29] showed that the effective antimicrobial activity of gentamicin loaded into liposomes involved fusion of the particles with the bacterial membrane, leading to its deformation. Further experiments are needed to better understand the interactions between the lipids in nanoformulations and the cellular membrane of microorganisms that promote drug diffusion.

The slower killing of the mucoid, resistant strain of *P. aeruginosa* than of the non-mucoid, susceptible strain by free as well as nanoencapsulated tobramycin can be explained by the additional time needed for outer membrane permeabilization by the drug, regardless of its method of preparation, and the subsequent delay in its reaching its intracellular target.

A previous study showed an immediate effect of tobramycin against most of the susceptible populations tested but not against the resistant population [30].

### 3.4. Anti-Biofilm Efficacy of Free and Nanoencapsulated Tobramycin

To test the influence of the lipid nanoparticles on tobramycin’s ability to kill sessile bacteria, biofilms of four *P. aeruginosa* strains were exposed to free and nanoencapsulated (SLN and NLC) tobramycin at antibiotic concentrations between 0 and 256 μg/mL. ATCC strain 27,853 and strain PAO1 were used as controls, and strains 056SJD (non-mucoid, tobramycin-susceptible) and 362VH (mucoid, tobramycin-resistant) as the *P. aeruginosa* CF isolates. All *P. aeruginosa* strains used in this experiment formed adequate biofilms (data not shown). The MIC and MBEC values of the four strains are shown in Table 3. Among the isolates susceptible to tobramycin, the MIC and MBEC values of the nanoencapsulated drug were slightly lower (1–2 logs) than those of the free drug. However, for the clinical isolate resistant to tobramycin, there were no differences in the MIC and MBEC values obtained with the nanoparticles and free tobramycin. The exception was NLC-tobramycin, in which the MBEC was slightly lower than the value obtained with the free form. The much higher MBEC vs. MIC values of both free and nanoencapsulated tobramycin likely reflected the interaction between the anionic mucopolysaccharide of the biofilm and the cationic aminoglycoside, such that the amount of free tobramycin available to act against the resident bacteria was limited [31]. Among the two types of nanoparticles (SLN and NLC), NLC were slightly more active than SLN (1 log) for all strains tested. Specifically, the concentrations of free tobramycin needed to completely eradicate the *P. aeruginosa* biofilm were 8–16 μg/mL (tobramycin-susceptible strains) and 32 μg/mL (tobramycin-resistant strain), but the effective NLC-tobramycin concentration was lower (2–4 μg/mL and 16 μg/mL, respectively). The better results obtained with the NLC formulation of tobramycin were in agreement with our previously published results showing that colistin-loaded NLCs were highly effective in biofilm eradication [20]. A modification of MBEC assay was performed to test the efficacy of NLC-tobramycin to prevent the biofilm formation [32]. For all the isolates tested, BPC (biofilm prevention concentration) values of NLC-tobramycin were identical to values for free tobramycin. Thus, whereas NLC-tobramycin was more effective than its free form in eradicating biofilms, both free and nanoencapsulated tobramycin did not show any differences on the prevention on biofilm formation.

Although the mechanisms underlying the efficacy of tobramycin in NLC are not fully understood, a role for charge distribution seems likely. Tobramycin loaded into NLC has a negative net charge because of the negatively charged nanoparticles, in contrast to the positive net charge of free tobramycin. The superior mucus penetration of negatively charged nanoparticles has been reported [33] and suggests the greater ability of NLC-tobramycin to penetrate the exopolyssacharide matrix surrounding the biofilm structure. Increased penetration would better allow tobramycin to reach its cellular target, in contrast to its free form. Alternatively, the fast-antimicrobial release reported by Pastor et al. [21] would ensure an initial antimicrobial concentration that is high enough to inhibit the biofilm growth of *P. aeruginosa*. Moreover, a sustained antimicrobial concentration higher than the MIC value would enable the eradication of surviving cells. However, further experiments are needed to determine which, if any, of our hypotheses is the correct one.

## 4. Conclusions

New antimicrobial formulations, such as lipid nanoparticles, can improve the transfer of antimicrobials to their sites of action, potentially allowing a dose reduction and therefore the avoidance of adverse side effects. Our study of planktonic cultures and biofilms of *P. aeruginosa* demonstrated that antimicrobial activity of tobramycin was not affected by nanoencapsulation. Thus, we found that nanoencapsulation of tobramycin did not improve its efficacy against planktonic *P. aeruginosa*. However, nanoencapsulation did improve its ability to eradicate *P. aeruginosa* biofilms. Given the key role of biofilms in respiratory infections of *P. aeruginosa* in CF patients, the results obtained in this study, and especially with NLC-tobramycin, may provide new options in the treatment of these infections, particularly taking into account the better distribution of antibiotics when inhaled as nanoparticles.

## Figures and Tables

**Table 1:** Bacterial strains used in this research. Strains SJD were isolated in Sant Joan de Déu Hospital and those being VH in the Hospital of Vall d’Hebrón. Abbreviations: Piper/Tz: Piperacillin/Tazobactam; Caz: Ceftazidime; Azt: Aztreonam; Imp: Imipenem; Mero: Meropenem; Gnt: Gentamicin; Tobra: Tobramycin; Amk: Amikacin; Col: Colistin; Cpfx: Ciprofloxacin. S: Susceptible; R: Resistant; I: Intermediate.

Source	Patient		Characteristics		Antibiotics									
Strain	Age	Gender	Mucoid	Hemolysis	PIPER/TZ	CAZ	AZT	IMP	MERO	GNT	TOBRA	AMK	COL	CPFX
PA 056 SJD	14	Male	–	ß	R	S	R	I	R	S	S	S	S	R
PA 086 SJD	13	Female	+	ß	S	S	S	S	S	R	R	s	s	S
PA 571.1 SJD	10	Male	+	–	S	S	S	S	S	S	S	S	S	S
PA 571.2 SJD	10	Male	+	–	S	S	S	S	S	S	S	S	S	S
PA 288 SJD	13	Male	–	–	S	S	R	R	R	I	S	S	S	S
PA 596 SJD	9	Male	–	ß	S	S	S	S	S	S	S	S	S	S
PA 666 SJD	13	Male	–	ß	S	S	S	S	S	S	S	S	S	R
PA 686 SJD	13	Male	–	ß	S	S	S	S	S	S	S	S	S	S
PA 744 SJD	14	Female	–	–	S	S	S	S	S	S	S	S	S	S
PA 668 SJD	2	Female	–	–	S	S	S	S	S	S	S	S	S	S
PA 721 SJD	7	Female	–	ß	S	S	S	S	S	S	S	S	S	S
PA 122 SJD	11	Male	+	ß	S	S	S	S	S	S	S	S	S	S
PA 788 SJD	7	Female	–	ß	S	S	S	S	S	R	S	S	S	S
PA 768 SJD	14	Male	–	ß	S	S	R	I	R	S	S	S	S	R
594 SJD	9	Male	+	ß	S	S	S	S	S	S	S	S	S	S
2881M SJD	13	Male	+	–	S	I	R	R	R	S	S	S	S	R
610M SJD	13	Male	+	–	S	S	R	R	R	S	S	S	S	R
610 SJD	13	Male	–	ß	S	S	R	I	R	S	S	S	S	R
805 SJD	15	Female	–	ß	S	S	S	S	S	R	S	S	S	S
555.1 SJD	7	Female	+	–	S	S	S	S	S	S	S	S	S	S
PA 417 VH	17	Female	–	ß	R	R	R	S	S	S	S	S	S	R
PA 362 VH	36	Male	+	ß	S	S	S	S	S	S	R	S	S	S
PA 684 VH	32	Male	–	–	S	S	I	R	R	S	S	I	S	I
PA 103 VH	29	Female	+	–	R	S	S	S	S	R	S	R	S	S
023 VH	15	Male	+	–	S	R	I	R	R		R	R	S	S
852 VH	17	Male	–	–	S	S	S	S	S	S	S	S	S	S
153 VH	17	Female	+	–	S	S	S	S	S	S	S	S	S	R
516 VH	20	Female	+	–	S	S	S	S	S	S	S	S	S	S
547 VH	15	Male	+	–	R	R	R	R	R	R	R	R	S	R
861 VH	23	Male	+	ß	S	S	S	S	S	S	S	S	S	S
639 VH	18	Male	+	–	S	S	S	S	S	S	S	S	S	R
897 VH	26	Female	–	–	S	S	S	S	S	S	S	S	S	I
697 VH	10	Female	–	ß	R	S	S	S	S	R	R	R	S	R
458 VH	32	Male	+	ß	S	S	S	R	R	R	S	R	S	R

**Table 2:** Characteristics of nanoparticle (TB tobramycin).

Formulation	Mean Size (nm)	PDI	Zeta-Potential (mV)	Percentage EE (Encapsulation Efficiency)
TB-SLN	302 ± 20.5	0.361 ± 0.02	−20.5 ± 6.09	ND
TB-NLC	254.05 ± 14.5	0.311 ± 0.01	−23.03 ± 2.76	93.15 ± 0.65

**Table 3:** Minimal biofilm eradication concentration (MBEC) and minimum inhibitory concentration (MIC) of free and NLC-encapsulated tobramycin. *P. aeruginosa* ATCC 27853 and strain PAO1 were used as controls. Strains 056SJD (non-mucoid, tobramycin-susceptible) and 362VH (mucoid, tobramycin-resistant) served as the *Pseudomonas*.

	ATCC 27853		PAO1		056SJD		362VH	
	MIC (μg/mL)	MBEC (μg/mL)	MIC (μg/mL)	MBEC (μg/mL)	MIC (μg/mL)	MBEC (μg/mL)	MIC (μg/mL)	MBEC (μg/mL)
Free Tobramicin	0.5	8	0.5	16	1	16	16	32
SLN-SDS-Tobramicin	0.25	4	0.25	8	0.5	8	16	32
NLC- Tobramicin	≤0.0625	2	0.25	4	0.25	4	16	16
